# Abnormalities in mitochondrial energy metabolism induced by cryopreservation negatively affect goat sperm motility

**DOI:** 10.3389/fvets.2024.1514362

**Published:** 2025-01-06

**Authors:** Shengqin Zang, Shuqi Zou, Xiangyi Chen, Bo Pan, Ao Ning, Jianpeng Qin, Yaozong Wei, Kunlin Du, Jiangfeng Ye, Qiuxia Liang, Yi Fang, Qiongla, Cirenlamu, Tianzeng Song, Guangbin Zhou

**Affiliations:** ^1^Farm Animal Genetic Resources Exploration and Innovation Key Laboratory of Sichuan Province, College of Animal Science and Technology, Sichuan Agricultural University, Chengdu, China; ^2^College of Life Science, Sichuan Agricultural University, Ya'an, Sichuan, China; ^3^Key Laboratory of Animal Production, Product Quality and Security, Ministry of Education, College of Animal Science and Technology, Jilin Agricultural University, Changchun, China; ^4^The Service Station of Agricultural and Animal, Husbandry Technical of Nyalam County, Shigatse, China; ^5^Institute of Animal Science, Xizang Academy of Agricultural and Animal Husbandry Science, Lhasa, China; ^6^Key Laboratory of Animal Genetics and Breeding on Xizang Plateau, Ministry of Agriculture and Rural Affairs, Lhasa, China

**Keywords:** goat sperm, cryopreservation, sperm motility, energy metabolism, metabolomics

## Abstract

The motility of sperm decreases following cryopreservation, which is closely associated with mitochondrial function. However, the alterations in mitochondrial metabolism after sperm freezing in goats remain unclear. This experiment aimed to investigate the impact of ultra-low temperature freezing on goat sperm’s mitochondrial energy metabolism and its potential correlation with sperm motility. The results revealed that goat sperm exhibited mitochondrial vacuolization, reduced matrix density, and significantly decreased levels of high-membrane potential mitochondria and adenosine triphosphate content, accompanied by a substantial increase in reactive oxygen species levels, ultimately leading to a significant decline in sperm viability. Further investigations unveiled that energy-related differential metabolites (capric acid, creatine, and D-glucosamine-6-phosphate) and differential metabolites with antioxidant effects (saikosaponin A, probucol, and cholesterol sulfate) were significantly downregulated. In addition, the activity of key rate-limiting enzymes involved in very long-chain fatty acid biosynthesis and *β*-oxidation—specifically acetyl-CoA carboxylase, fatty acid synthase, and carnitine palmitoyltransferase I related to capric acid metabolism—was considerably reduced. Furthermore, supplementation of differential metabolite capric acid (500 μM) significantly enhanced the motility of frozen–thawed goat sperm. These findings indicated that the mitochondrial ultrastructure of goat sperm is damaged and energy metabolism becomes abnormal after cryopreservation, potentially affecting sperm viability. The addition of different metabolites such as capric acid to the freezing extender can alleviate the decrease in sperm motility induced by cryopreservation.

## 1 Introduction

Semen cryopreservation technology has the potential to overcome the limitations of time and space, facilitating long-distance transportation of animal semen and accelerating the global adoption and application of modern biotechnology such as artificial insemination and *in vitro* fertilization (1). However, it is worth noting that post-thaw sperm motility is significantly lower compared to fresh sperm: 39.47% vs. 87.33% in pigs (2, 3), 36.0% vs. 83.4% in horses (4, 5), 37.12% vs. 90.30% in cattle (6, 7), 37.60% vs. 82.18% in sheep (8, 9), and 23.3% vs. 80.0% in goats (10, 11). Previous studies have indicated that decreased sperm motility after freezing is associated with mitochondrial damage (12–14). Given that mitochondria play a key role in energy metabolism, which is closely related to sperm motility (15). Thus, sperm mitochondria damage could be tightly linked to decline of sperm motility (16, 17). It has been reported that the mitochondrial permeability transition pore, a transmembrane protein on the inner mitochondrial membrane (IMM), usually remains closed (18). However, sperm cryopreservation tends to cause the formation and prolonged opening of the mitochondrial permeability transition pore, which leads to the disruption of the energy supply and causes mitochondrial damage (19, 20). Apparently, there is an inevitable connection between the energy metabolism disruption induced by mitochondrial dysfunction and the decline in sperm motility; however, the exact mechanism is still unclear. Therefore, investigating the characteristics of mitochondrial energy metabolism following sperm cryopreservation can provide valuable insights into understanding the mechanisms underlying reduced sperm motility and exploring potential mitigation strategies.

After sperm cryopreservation, the mitochondria primarily exhibit ultrastructural damage, a reduction in adenosine triphosphate (ATP) content, decreased mitochondrial membrane potential (MMP), and abnormal opening of the mitochondrial membrane permeability transition pore. This is also accompanied by the production of a large amount of reactive oxygen species (ROS), especially in complexes I and III of the mitochondrial electron transport chain (ETC) (21, 22). Excessive accumulation of ROS leads to oxidative stress, which damages the mitochondrial membrane and its function, leading to a self-perpetuating cycle of ROS generation (15, 23). Previous studies have demonstrated that freezing results in mitochondrial ultrastructural damage in human sperm and significant alterations in the citrate cycle (TCA cycle) in mitochondria (24). However, further investigation is needed to explore how mitochondrial metabolism changes after sperm cryopreservation in goats and its impact on sperm viability.

As metabolomic technology continues to advance, there has been a notable increase in studies targeting metabolites in the frozen–thawed sperm of domestic animals. An analysis of the results of these existing studies revealed that there are differences in the sperm metabolomic profiles of different species (such as Dorper sheep and bull) (25, 26) or breeds (such as Yunshang black goat and Guanzhong dairy goat) (27, 28). This suggests that exploring the key differential metabolites specific to different breeds may enhance the effectiveness of sperm cryopreservation. However, the changes in energy metabolism in semen from the Chengdu brown goat (CBG), a breed recognized under China’s National Product of Geographical Indication, after freezing, remains unclear. Therefore, this study aims to investigate (1) the impact of cryopreservation on mitochondrial ultrastructure, function, and sperm motility in CBG, (2) the effects of ultra-low temperature freezing on energy metabolism in CBG sperm and identification of differential metabolites, and (3) the influence of these differential metabolites on the motility of frozen–thawed CBG sperm.

## 2 Materials and methods

Unless otherwise noted, all chemicals were purchased from Sigma-Aldrich (St. Louis, MO, USA). All experimental procedures were conducted in strict accordance with the regulations of the Animal Ethics and Welfare Committee (AEWC) of Sichuan Agricultural University, China (Approval code: AEWC2016, 6 January 2016).

### 2.1 Animal semen collection and quality assessment

We collected semen at Xilingxue Agricultural Development Co., Ltd. (103°17′E, 30°35’N) in Chengdu, Sichuan Province, China, during the breeding season (August–November) in 2023. A total of 10 healthy goats of normal breeding age (2–4 years old) were selected, and each male goat was individually housed and managed. All male goats had free access to water and feed. Semen was collected from the male goats using an artificial vagina method (with water temperature controlled between 40 and 42°C). Collections were performed once a week for 12 weeks, with semen samples taken from five male goats each time. The semen was collected using disposable centrifuge tubes, and the volume was recorded each time. First, the color and viscosity of the semen were observed visually. Then, the collected semen was diluted with a base extender, wrapped in a cotton gauze, and transported in a foam box (18–20°C) to the laboratory within 1 h.

A 3 μL aliquot of diluted semen was transferred into a pre-heated (37°C) disposable sperm test plate. Computer-assisted semen analysis (CASA) (AndroVision®, Minitube, Germany) was used to select the goat species in the software, and the relevant parameters were analyzed and recorded at 200x magnification. For each sample, five fields were counted, and at least 2,500 sperm were analyzed. The CASA system parameters were set as described in published article (8), including curvilinear velocity (VCL; μm/s), velocity of straight line (VSL; μm/s), velocity of average path (VAP; μm/s), beat cross-frequency (BCF; HZ); radius: sperm path radioactivity coefficient. Briefly, sperm trajectory motion were continuously captured within 0.5 s using the following parameters, in situ motility (VSL < 24.0000, VCL < 48.0000), circular motion (radius > 9.00 and radius < 90.00 and rotation >0.70) and slow motion (VCL < 120.0000), respectively. The semen samples used in this study had to meet the following criteria: volume ranging from 0.7 mL to 2 mL, a minimum sperm density of 2.5 × 10^9^ sperm/mL, and total motility of at least 70%.

### 2.2 Semen dilution, freezing, and thawing

The semen from each goat after passing the quality test was equally divided into four groups: fresh and frozen groups treated with commercial diluent (CD) and fresh and frozen groups treated with homemade diluent. The CD for the goat was divided into liquid I and liquid II (Beijing Tianyuan Aorui Biotechnology Co., Ltd., China). The commercial fresh group was diluted with liquid I (1:19). When the CD is used as the cryopreservation diluent, 25% (v:v) fresh yolk solution should be added to both liquid I and liquid II before use. In brief, the homemade base extender composition included 19.98 mM Tris (T8060, Solarbio, China), 4.67 mM glucose, 7.18 mM citric acid (C8610, Solarbio, China), 5.84 mM sucrose, and 100,000 IU each of penicillin and streptomycin. This solution was used to dilute fresh sperm at a ratio of 1:19, with 100 mL of dilution solution. Liquid I was prepared by adding 20% egg yolk (v:v) to the homemade base extender. Liquid II was then prepared by adding 6% glycerol (v:v) and 0.6% Equex STM paste (v:v) to liquid I (13,560/0008, Minitube, Germany).

**Figure 1:**
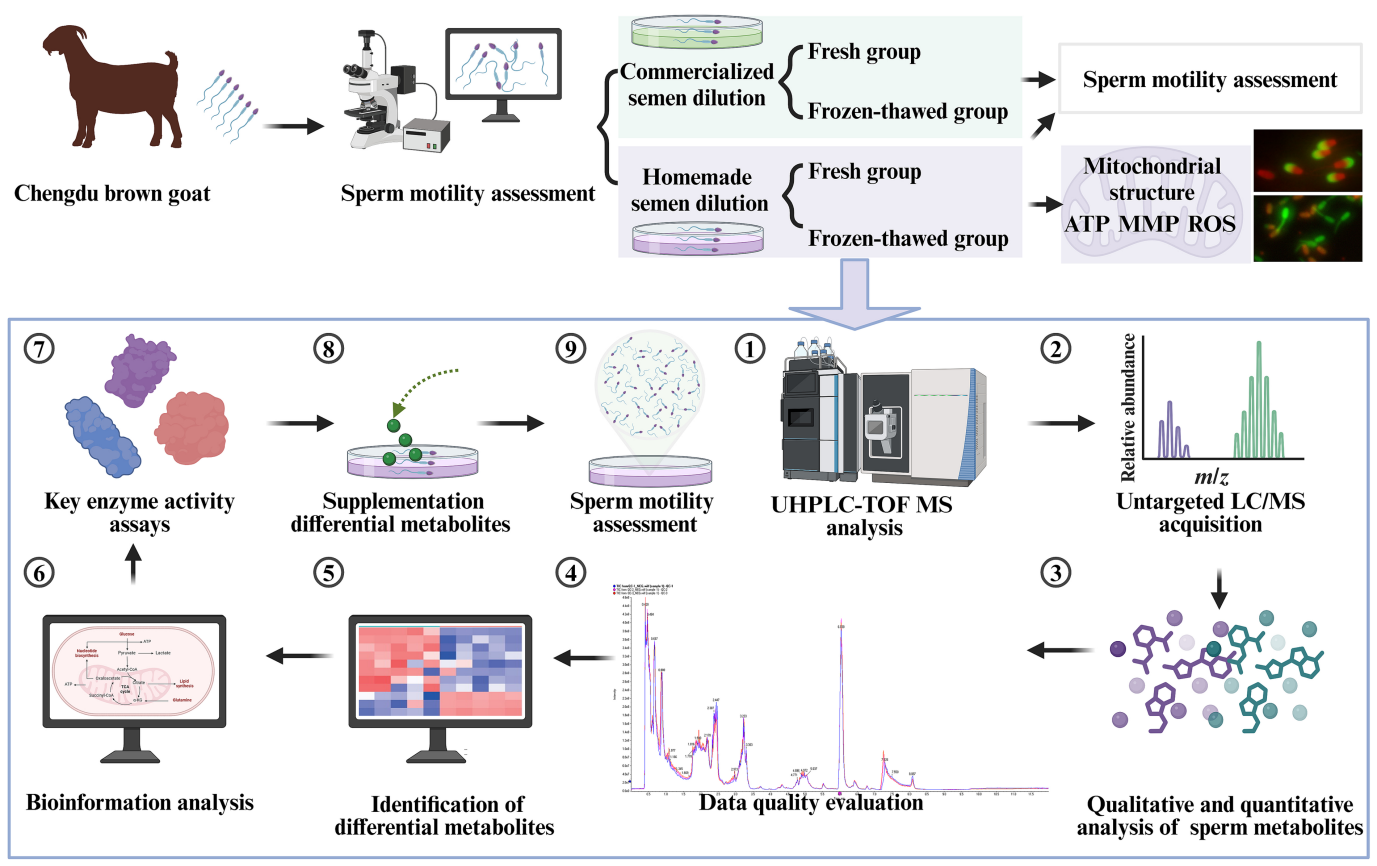
Schematic diagram of the experimental design. UHPLC-Q-TOF-MS is an ultra-high performance liquid chromatography quadrupole time-of-flight mass spectrometer (created in BioRender.com).

Semen freezing was performed using a two-step dilution method. The specific procedure was as follows: Cryopreservation dilution liquids I and II containing sperm were mixed homogeneously (1:9:10) at isothermal and equal volumes; then, the semen samples were packed in 0.25 mL plastic straws (1.25 × 10^8^ sperm/mL), which was sealed with polyvinyl alcohol powder, wrapped in 8 layers of cotton gauze, and placed in a refrigerator at 4°C for cooling and equilibration for 1.5 to 2 h. The plastic straws were placed 4 cm above liquid nitrogen for 10 min, and the temperature was kept at −130°C, then plunged into liquid nitrogen (− 196°C), and kept for 7 days. The straws were thawed in a water bath at 37°C for 30 s before use. The qualified semen, with sperm motility above 0.35, was first centrifuged at 4,000 rpm and 4°C for 5 min to remove the supernatant. Then, it was resuspended in phosphate buffer saline (PBS) without calcium and magnesium, washed three times, and diluted to a concentration of 1.25 × 10^8^ sperm/mL, The diluted semen was incubated at 37°C for 1 h, followed by centrifugation again (4,000 rpm, 4°C, 5 min) in preparation for the next step of metabolomics analysis. The flowchart of the experimental design is shown in Figure 1.

### 2.3 Sperm ATP content, ROS levels, and MMP assays

The Enhanced ATP Assay Kit (S0027, Beyotime, China) was used for the analysis. Before the analysis, the thawed semen was diluted to 1.6 × 10^6^ sperm/mL, washed three times with calcium and magnesium-free PBS, and centrifuged at 4000 rpm for 5 min at 4°C each time; after discarding the supernatant, the precipitate was left. After each group was added with 200 μL lysate, repeated blowing was performed to lyse the cells (placed on ice). After lysis, the cells were centrifuged at 4°C 12000 *g*, and the supernatant was taken and used for the determination of subsequent tests. The ATP standard solution was diluted to concentrations of 0.01, 0.03, 0.1, 0.3, 1, and 3 μM using ATP assay solution. The ATP assay reagent was then diluted with the ATP assay reagent diluent at a ratio of 1:4. Next, 100 μL of ATP assay working solution was added to the assay wells and left at room temperature for 3–5 min to allow for the consumption of any background ATP. The RLU values were determined using a luminometer (Varioskan LUX, Thermo, USA), and ATP content was calculated.

A volume of 900 μL of fresh semen was injected into a clean EP tube. A volume of 100 μL of Rosup reagent from the reactive oxygen detection kit (CA1410, Solarbio, China) was added to the test tube and then incubated for 20 min at 37°C in an incubator as a positive control sample to be tested. The volume of 2′,7’-Dichlorodihydrofluorescein diacetate (DCFH-DA) working solution (200 μL/tube) was calculated according to the number of samples to be tested, and the DCFH-DA probe was diluted with sterile PBS buffer at a ratio of 1:3000. After that, 200 μL of each semen to be tested was mixed with an equal volume of working solution and then incubated at 37°C for 20 min, during which the samples were inverted several times every 5 min to make full contact between the semen and the probe. Another sample without a probe was set as a blank control. After incubation, the samples were centrifuged (4,000 rpm, 37°C, 5 min), the supernatant was discarded and washed twice with PBS, and 500 μL of PBS was added to the precipitate to be detected by flow cytometry.

Next, 1 mg of JC-1 dry powder (J8030, Solarbio, China) was added to 1 mL of dimethyl sulfoxide (DMSO) and mixed well to form a storage solution. PBS was used for the dilution of JC-1 stock solution to create a final concentration of 250 μg/mL (working solution). An equal volume of semen and working solution was taken and mixed well by gently blowing followed by incubation at 37°C for 20 min and centrifugation (4,000 rpm, 4°C, 5 min). After that, the supernatant was discarded and precipitation was washed twice with PBS (4,000 rpm, 4°C, 5 min), and finally, 600 μL of PBS was added. For the blank control, a sample without JC-1 treatment was used. A Canto II flow cytometer (Becton Dickinson, Franklin Lakes, NJ, USA) was used for the evaluation of samples by setting a total of 10,000 events per sample, and then, data were processed using FlowJo software (Becton Dickinson).

### 2.4 The ultrastructure analyses of sperm mitochondria, acrosome, and plasma membrane by transmission electron microscopy

Fresh group sperm were centrifuged directly (1,000 rpm, 4°C, 5 min), and the supernatant was discarded. The spermatozoa of the frozen group were thawed and washed three times with calcium and magnesium-free PBS (1,000 rpm, 4°C, 5 min), and the supernatant was discarded after the final time to leave the precipitate. The cells were resuspended by slowly adding 1:5 (3% glutaraldehyde: 0.1 mol/L PBS buffer) diluted fixative along the wall of the tubes with a pipette and left to stand for 5 min at 4°C. High-speed centrifugation (12,000 rpm, 4°C, 10 min) was performed, the supernatant was discarded, and the precipitate was retained. Then, 3% glutaraldehyde fixative (3%, Sinopharm Chemical Reagent Co., Ltd., China) was again added along the wall of the tube (not to be blown out of the cells) and stored at 4°C. The collected samples were fixed, dehydrated, permeabilized, embedded, sectioned, and stained, and then, the mitochondrial ultrastructure of spermatozoa was observed using transmission electron microscopy (JEM-1400FLASH, JEOL, Japan).

### 2.5 Acrosome integrity and membrane integrity assays

Fluorescein isothiocyanate conjugated peanut agglutinin (FITC-PNA; Sigma, L7381) and propidium iodide (PI) were used to detect sperm acrosome integrity according to the manufacturer’s instruction. In brief, after washing three times with PBS, 980 μL of sperm suspension was stained with 20 μL of FITC-PNA stock solution (20 μg/mL) and 5 μL of propidium iodide (PI) stock solution (20 μg/mL) for 25 min in the dark. Aliquots of the stained suspensions were analyzed using the fluorescence microscope.

Membrane integrity was measured using the LIVE/DEAD™ Sperm Viability Kit (L-7011; Thermo Fisher Scientific) according to the manufacturer’s instructions. In brief, the goat sperm samples were stained with SYBR-14/PI. The staining was analyzed using fluorescence microscopy with excitation/emission = 485/517 nm for SYBR-14 fluorescence and excitation/emission = 586/617 nm for PI fluorescence. A total of 2,896 sperm were analyzed.

### 2.6 Metabolomics analysis of goat sperm

The sperm precipitation was followed by thawing at 4°C, and then 800 μL of cold methanol/acetonitrile (1:1) was used to remove proteins and extract metabolites. The mixture was collected into a new centrifuge tube and centrifuged at 14000 *g* for 20 min to collect the supernatant, and then the supernatant was dried in a vacuum. For liquid chromatography–tandem mass spectrometry (LC–MS) analysis, the samples were re-dissolved in 100 μL acetonitrile/water (1:1, v/v) solvent and centrifuged at 14000 *g* at 4°C for 15 min; then the supernatant was injected.

Analysis was performed using ultra-high-performance liquid chromatography (UHPLC) (1,290 Infinity LC, Agilent Technologies; Shanghai Applied Protein Technology Co., Ltd.; Shanghai, China). For hydrophilic interaction liquid chromatography (HILIC) separation, the samples were analyzed using a 2.1 mm × 100 mm ACQUIY UPLC BEH Amide 1.7 μm column (waters, Ireland). In both positive and negative modes of electrospray ionization (ESI), the mobile phase contained 25 mM ammonium acetate +25 mM ammonium hydroxide in water (labeled A) and acetonitrile (labeled B). The gradient was 95% B for 0.5 min, linearly reduced to 65% in 6.5 min, reduced to 40% in 1 min and kept for 1 min, and then increased to 95% in 0.1 min, with a 3-min re-equilibration period employed. Gradients were at a flow rate of 0.5 mL/min, and column temperatures were kept constant at 25°C. A 2 μL aliquot of each sample was injected.

The mass spectrometry analysis was performed using a quadrupole time-of-flight mass spectrometer (Q-TOF) (AB SCIEX TripleTOF 6,600; Shanghai Applied Protein Technology Co., Ltd.; Shanghai, China). The conditions for the ESI source were set as follows: Ion Source Gas 1 and 2 were both set at 60, the curtain gas was set to 30, the source temperature was set to 600°C, and the ion spray voltage floating (ISVF) was set to ±5,500 V. During MS-only acquisition, the instrument was programmed to acquire data over the m/z range of 60–1,000 Da, with an accumulation time of 0.20s/spectrum for TOF MS scans. For auto MS/MS acquisition, the instrument was set to acquire data over the m/z range of 25–1,000 Da, with an accumulation time of 0.05 s/spectrum for production scans. The production scan was obtained using information-dependent acquisition (IDA) with a high-sensitivity mode selected. The parameters for IDA were as follows: The collision energy (CE) was fixed at 35 V with a ± 15 eV range, the declustering potential (DP) was set to 60 V (+) and − 60 V (−), and isotopes within 4 Da were excluded. In addition, 10 candidate ions were monitored per cycle.

### 2.7 Metabolomics data processing and analysis

Raw MS data were converted to MzXML files using ProteoWizard MSConvert before free import was available in the XCMS software. For peak pickup, the following parameters were used: centWave m/z = 10 ppm; peak width = c (10, 60), and prefilter = c (10, 100). Peak grouping was performed with the following parameters: bw = 5, mzwid = 0.025, and minfrac = 0.5. The Collection of Algorithms of Metabolite Profile Annotation (CAMERA) was used for isotopes and adducts. Only variables with more than 50% non-zero measurements had at least one set of values that remained constant in the extracted ion features. Compound identification accuracy was assessed by comparing the m/z values (<10 ppm) of metabolites and MS/MS spectra with an in-house database (Shanghai Applied Protein Technology), which includes authentic standards for reliable identification.

Statistical analyses were performed, and data normality was assessed using SPSS (v.27.0, IBM, Chicago, IL, USA) with the Shapiro–Wilk test. After normalization, the processed data were analyzed using the R package (ropls), in which processed multivariate data analyses were carried out using overall sample principal component analysis (PCA) and orthogonal partial least squares-discriminant analysis (OPLS-DA) to differentiate the overall differences in metabolic profiles between the groups and to identify the metabolites that differed between the before and after semen freezing in CBG. To prevent model overfitting, we used a 7-fold cross-validation and response alignment test to assess the robustness of the model. Variable importance in the projection (VIP) obtained from the OPLS-DA model can be used to measure the strength and explanatory power of the influence of the expression pattern of each metabolite on the classification and discrimination of each group of samples and to mine biologically significant differential metabolites. Fold multiplicity of variation and VIP > 1, with *p* < 0.05, were used to screen for significantly varying metabolites. Student’s *t*-test was applied to determine the significance of differences between fresh and frozen groups. All metabolite identifications were performed using the in-house database (Shanghai Applied Protein Technology).

### 2.8 Acetyl coenzyme a carboxylase (ACACA), fatty acid synthase (FASN), and carnitine palmitoyltransferase I (CPT-1) activity assays

ACACA activity was measured according to the instructions of the assay kit (KTB1261, Abbkine, China). Briefly, fresh sperm and frozen–thawed goat sperm were washed with pre-cooled PBS. Briefly, frozen–thawed goat sperm were washed three times with pre-cooled PBS to remove extra homemade extender and then centrifuged to discard the supernatant (4,000 rpm, 4°C, 5 min). One milliliter of extraction buffer from the kit was added, and the sperm were broken by ultrasonic waves in an ice bath, with 30 cycles (200 W, ultrasonic waves for 3 s at 7 s intervals for a total time of 5 min). The mixture was then centrifuged (8,000 *g*, 4°C, 10 min), and the supernatant was collected and placed on ice to be measured. Then, the protein concentration of the samples was determined using a BCA protein assay kit (PC0020, Solarbio, China). Detection was carried out via a microplate reader with the wavelength parameter set to 660 nm. The ACACA activity was calculated from the sample protein concentration.

FASN activity was carried out according to the manufacturer’s instructions for the assay kit (BC0555, Solarbio, China). In short, 1 mL of the extract from the kit was added to the prepared sperm precipitate, broken by ultrasonic waves in an ice bath, with 30 cycles (power 300 W, ultrasonic waves for 3 s, intervals of 9 s, total time 5 min). The mixture was then centrifuged (12,000 *g*, 4°C, 20 min), and the protein concentration was detected after the supernatant was taken. Detection was performed via a microplate reader with the wavelength parameter set to 340 nm. The FASN activity was calculated according to the sample protein concentration.

CPT-1 activity was assayed according to the kit instructions (ml076617, Shanghai Enzyme-linked Biotechnology Co., Ltd., China). The wavelength setting parameter of the microplate reader was 412 nm. The CPT-1 activity was calculated by sample protein concentration.

### 2.9 Treatment of goat semen diluent with varying concentrations of capric acid

Different concentrations of capric acid were added to homemade semen diluents I and II, respectively, and divided into the following groups: fresh group and capric acid frozen–thawed group treated with different concentrations (0 μM, 125 μM, 250 μM, 500 μM, and 1,000 μM groups), incubated together at room temperature for 1 h, and then frozen by liquid nitrogen fumigation. Fresh and frozen–thawed groups (0 μM) were used as control groups, after at least 2 weeks of preservation in liquid nitrogen. Then, the motility of thawed sperm was evaluated according to the steps described in Section 2.3 of the Materials and Methods section.

### 2.10 Statistical analysis

All experiments were repeated at least three times, all raw data were counted and organized using Excel 2019 (Microsoft, USA). Statistical analysis was performed using SPSS (v.27.0, IBM, Chicago, IL, USA). Student’s *t*-test was performed to compare the two groups. A one-way analysis of variance (ANOVA) followed by *post-hoc* Fisher’s least significant difference (LSD) test was used to compare more than two groups. All data were expressed as mean ± standard error (mean ± SEM). Graphs were made jointly using GraphPad Prism (v.5.0, GraphPad Software LLC, USA) and Adobe Illustrator 2020 (Adobe Inc., USA). A *p*-value of < 0.05 indicates a statistical difference.

## 3 Results

### 3.1 Reduced motility of goat sperm following cryopreservation

**Figure 2:**
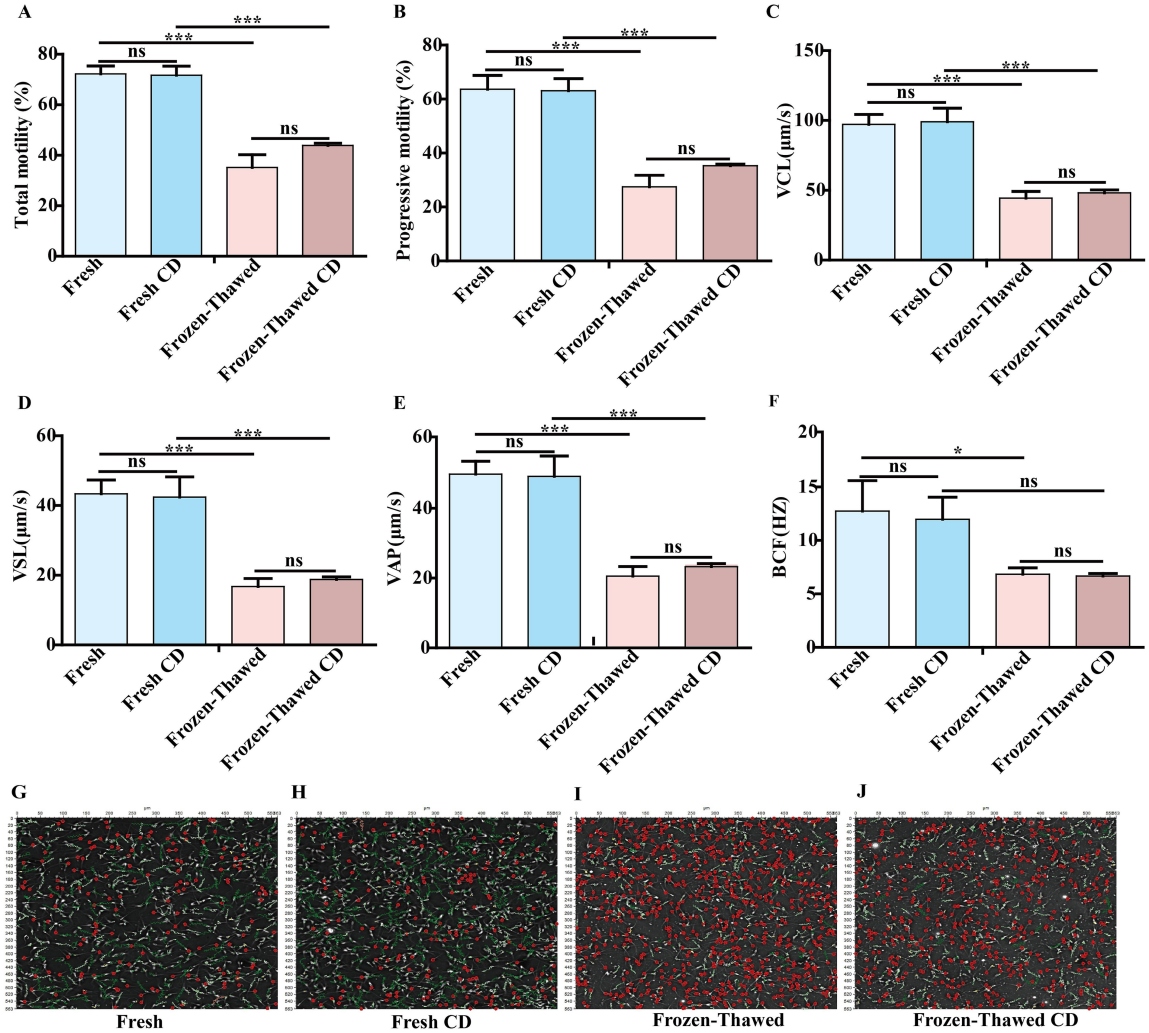
Comparison of the effect of homemade dilutions and commercial dilutions (CD) on goat semen freezing. **(A)** Total motility. **(B)** Progressive motility. **(C)** Velocity of trajectory curve (VCL; μm/s). **(D)** Velocity of straight line (VSL; μm/s). **(E)** Velocity of average path (VAP; μm/s) **(F)** Beat cross-frequency (BCF; HZ). Sperm movement trajectories of the fresh group: **(G)** The fresh group of homemade diluents is indicated by Fresh. **(H)** The fresh group of CD is indicated by Fresh CD. Movement trajectories of frozen–thawed spermatozoa: **(I)** The frozen group with homemade diluents is indicated by Frozen–thawed. **(J)** The frozen group with CD is indicated by Frozen–thawed CD. Orange trajectory: circular motion; dark green trajectory: fast motion; bright green motion trajectory: slow motion; yellow motion trajectory: *in situ* motion; red circle: inactive. Asterisks indicate *p* < 0.05 (*) and *p* < 0.001 (***), and “ns” indicates a non-significant difference.

After ultra-low temperature freezing, the total motility (72.21 ± 3.14% vs. 35.15 ± 5.05%, Figure 2A), progressive motility (63.65 ± 5.13% vs. 27.49 ± 4.31%, Figure 2B), VCL (97.16 ± 7.21 vs. 44.45 ± 4.77%, Figure 2C), VSL (43.36 ± 3.99 vs. 16.77 ± 2.30, Figure 2D), VAP (49.75 ± 3.73 vs. 20.57 ± 2.74, Figure 2E), and BCF (12.74 ± 2.86 vs. 6.85 ± 0.60, Figure 2F) of goat sperm were significantly reduced compared to the fresh group when using homemade dilutions (*p* < 0.05). Similarly, when a commercial diluent was used, the total motility (71.73 ± 3.59% vs. 43.91 ± 0.87%, Figure 2A), progressive motility (63.12 ± 4.48% vs. 35.30 ± 0.58%, Figure 2B), VCL (99.11 ± 9.73 vs. 48.13 ± 2.18, Figure 2C), VSL (42.44 ± 5.78 vs. 18.77 ± 0.76, Figure 2D), and VAP (49.14 ± 5.81 vs. 23.29 ± 0.91, Figure 2E) of goat sperm showed significant decrease after ultra-low temperature freezing compared to the fresh group (*p* < 0.05). However, there was no significant difference in BCF (11.97 ± 2.07 vs. 6.67 ± 0.35, Figure 2F) (*p* > 0.05). Furthermore, an analysis of the movement trajectories of fresh sperm diluted with homemade and commercial diluent showed no significant differences (Fresh vs. Fresh CD) (Figures 2G,H). Similarly, when sperm was diluted with homemade and commercial diluent before cryopreservation, there were also no significant differences in the movement trajectories for frozen-thawed sperm (Frozen-thawed vs. Frozen-thawed CD) (Figures 2I,J). These findings suggest both our homemade diluent and the commercial diluent can be used interchangeably for treating and freezing CBG semen without affecting sperm motility or other parameters in either the fresh or frozen–thawed groups (Figures 2A,B) (*p* > 0.05). Considering factors such as timeliness and cost, we chose to use our homemade diluent for subsequent experiments.

### 3.2 The cryogenic freezing process induces structural and functional impairments in the mitochondrial of goat sperm

**Figure 3:**
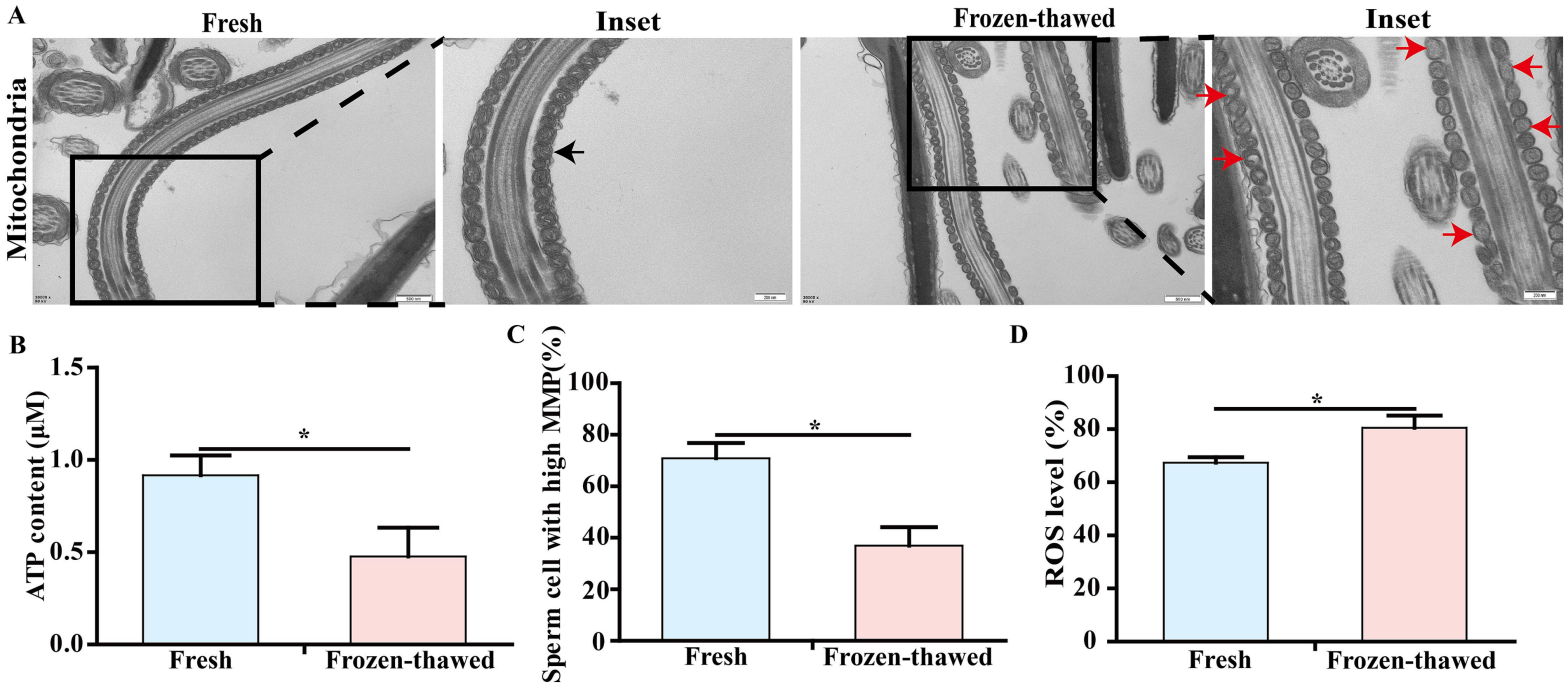
Effects of cryopreservation on the ultrastructure and ATP content of mitochondria, mitochondrial membrane potential (MMP), and ROS level in sperm of goat. **(A)** Transmission electron microscopy of sperm mitochondria, scale bar = 500 nm; local magnification scale bar = 200 nm; black arrows represent normal mitochondrial structure; red arrows represent mitochondrial structural vesicles destroyed, vacuoles, or mitochondrial matrix density reduced. **(B)** ATP content assay. **(C)** MMP assay. **(D)** ROS level assay. ‘*’ indicates statistical differences, *p* < 0.05.

Since mitochondria are closely associated with sperm viability, we subsequently investigated the mitochondrial ultrastructure and function of sperm. Our findings revealed that frozen CBG sperm exhibited vacuolization in the mitochondrial vesicles, as well as a reduction in the density of the mitochondrial matrix (Figure 3A), indicating impaired mitochondrial ultrastructure. Following ultra-low temperature freezing, there was a significant decrease in ATP content (0.92 ± 0.11 vs. 0.40 ± 0.17, Figure 3B), high-MMP ratio (70.83 ± 5.97% vs. 36.97 ± 7.13%, Figure 3C), and ROS level (67.37 ± 1.22% vs. 80.57 ± 2.64%, Figure 3D) of sperm compared to the fresh group (*p* < 0.05).

### 3.3 Cryopreservation compromises acrosome integrity and membrane integrity of goat sperm

**Figure 4:**
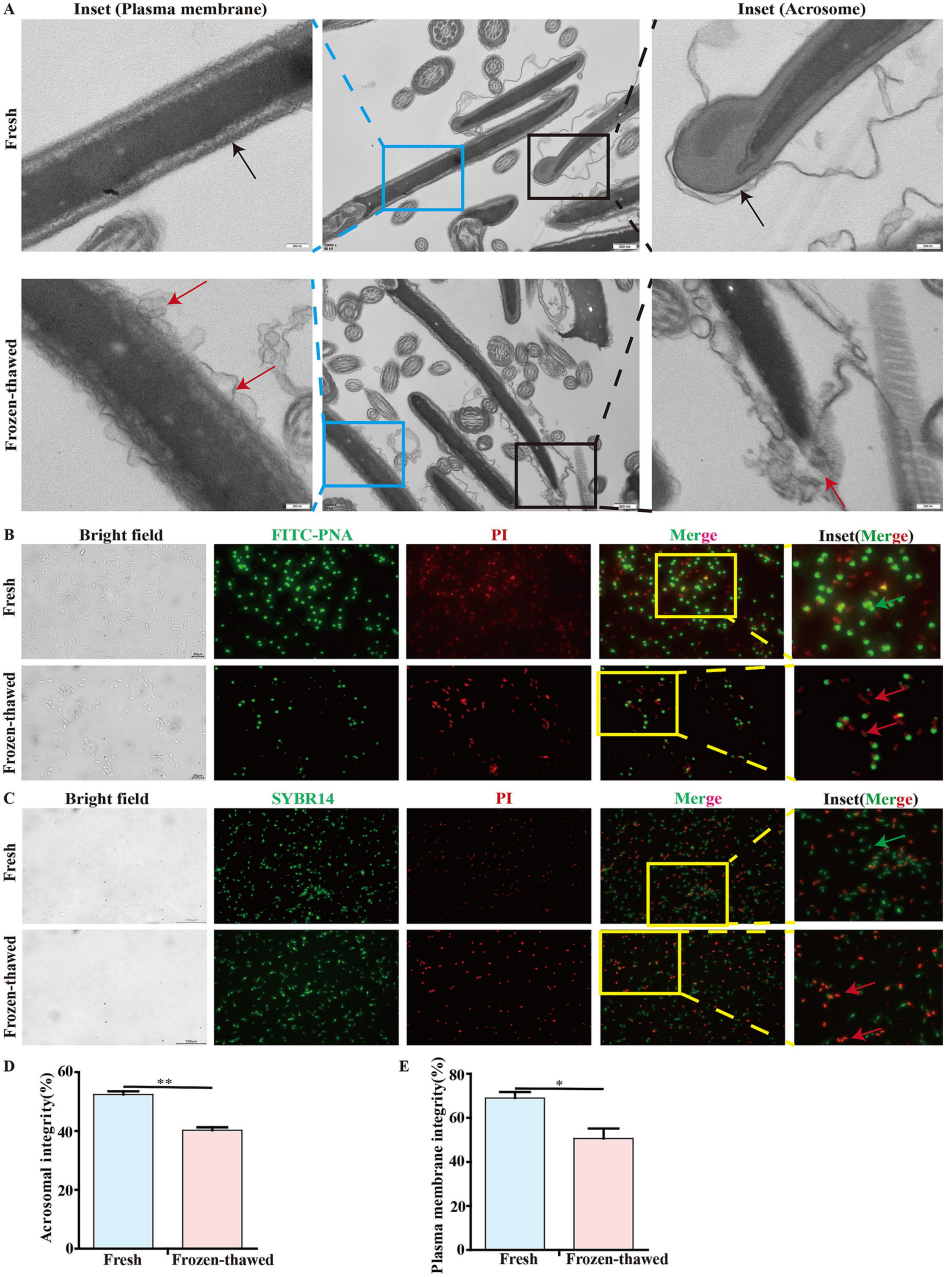
Effect of cryopreservation on the acrosome integrity and membrane integrity of goat sperm. **(A)** Detection of the ultrastructure of sperm plasma membrane and acrosome by transmission electron microscopy. The black box indicates the acrosome, and the blue box indicates the plasma membrane. In these two enlarged images, the black arrows represent the integrity of the acrosome and the integrity of the plasma membrane, respectively; the red arrows indicate the detachment of the acrosome and the damage to the plasma membrane, respectively. Scale bar = 500 nm; local magnification scale bar = 200 nm. **(B,C)** Detection of acrosome integrity and plasma membrane integrity of goat sperm using FITC-PNA/PI and SBYR14/PI fluorescent staining methods. The yellow box indicates the area selected for enlargement. In the detection of acrosome integrity, scale bar = 20 μm. In the detection of the plasma membrane, scale bar = 100 μm. In these two enlarged images, the green arrows represent sperm with intact acrosomes and plasma membranes, respectively, while the red arrows represent sperm with acrosome loss or damage and plasma membrane injury, respectively. **(D,E)** The bar chart represents the statistical analysis of the percentage of fluorescent staining results for sperm acrosome integrity and membrane integrity. Asterisks indicate *p* < 0.05 (*) and *p* < 0.01 (**).

To elucidate the effect of cryopreservation on the integrity of the acrosome and plasma membrane of goat sperm, we observed the ultrastructure of the sperm acrosome and plasma membrane using transmission electron microscopy, respectively. The results showed that sperm exhibited acrosomal detachment and plasma membrane damage after freezing (Figure 4A). In addition, we reassessed the sperm acrosome and plasma membrane integrity by fluorescence staining that showed a significant decrease in acrosomal integrity of sperm (40.27 ± 0.99% vs. 52.41 ± 1.11%, *p* < 0.01) (Figures 4B,D) and the integrity of sperm plasma membrane (50.62 ± 4.56% vs. 69.04 ± 2.71%, *p* < 0.05) (Figures 4C,E), respectively, in frozen–thawed group compared to the fresh group.

### 3.4 Metabolomic analysis of goat sperm pre- and post-cryopreservation

**Figure 5:**
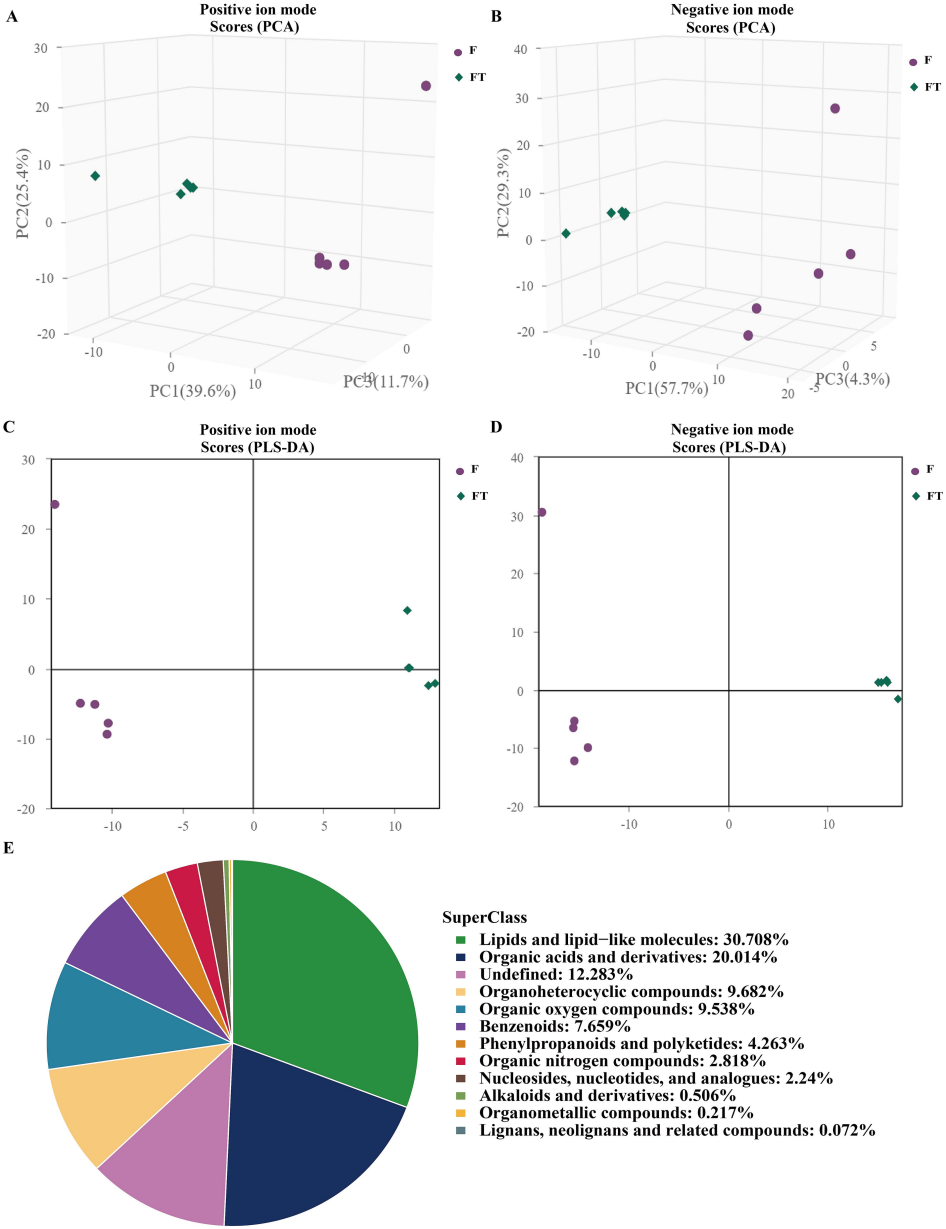
Graphs of multivariate statistical analyses and chemical classification of metabolites identified in goat sperm. **(A,B)** Graphs of PCA scores in positive and negative ion modes. **(C,D)** Graphs of PLS-DA scores in positive and negative ion modes. **(E)** Chemical classification of identified metabolites. (1) The horizontal coordinate t[1] in the graph represents principal component 1, and the vertical coordinate t[2] represents principal component. (2) The dots of the same color indicate the individual biological replicates within the group, and the distribution status of the dots reflects the degree of inter- and intra-group variability.

To further evaluate the impact of cryopreservation-induced mitochondrial damage on mitochondrial metabolism, we performed untargeted metabolomics analysis on pre- and post-freezing sperm samples. Our findings demonstrated a clear distinction in metabolite profiles between the fresh and frozen groups (Figures 5A–D), with validation provided by PLS-DA and OPLS-DA analysis models (Tables 1, 2). In total, we identified a total of 1,384 metabolites in goat sperm samples before and after freezing (772 in positive ion mode and 612 in negative ion mode). These metabolites were categorized based on their chemical composition into lipids and lipid-like molecules (30.708%), organic acids and derivatives (20.014%), undefined metabolites (12.283%), organoheterocyclic compounds (9.682%), and organic oxygen compounds (9.538%) (Figure 5E).

**Table 1:** PLS-DA validity.

Sample groups	Ion mode	A	*R*^2^*X*(cum)	*R*^2^*Y*(cum)	*Q*^2^(cum)
FT vs. F	Positive	1	0.646	0.997	0.971
	Negative	1	0.87	0.999	0.994

A: denotes the main score.

*R2X: denotes the explanation rate of the model to the X variable.*

*R2Y: denotes the explanation rate of the model to the Y variable.*

*Q2: denotes the predictive ability of the model.*

**Table 2:** OPLS-DA validity.

Sample groups	Ion mode	*A*	*R*^2^*X*(cum)	*R*^2^*Y*(cum)	*Q*^2^(cum)
FT vs. F	Positive	1	0.646	0.997	0.996
	Negative	1	0.87	0.999	0.997

A: denotes the main score.

*R2X: denotes the explanation rate of the model to the X variable.*

*R2Y: denotes the explanation rate of the model to the Y variable.*

*Q2: denotes the predictive ability of the model.*

**Figure 6:**
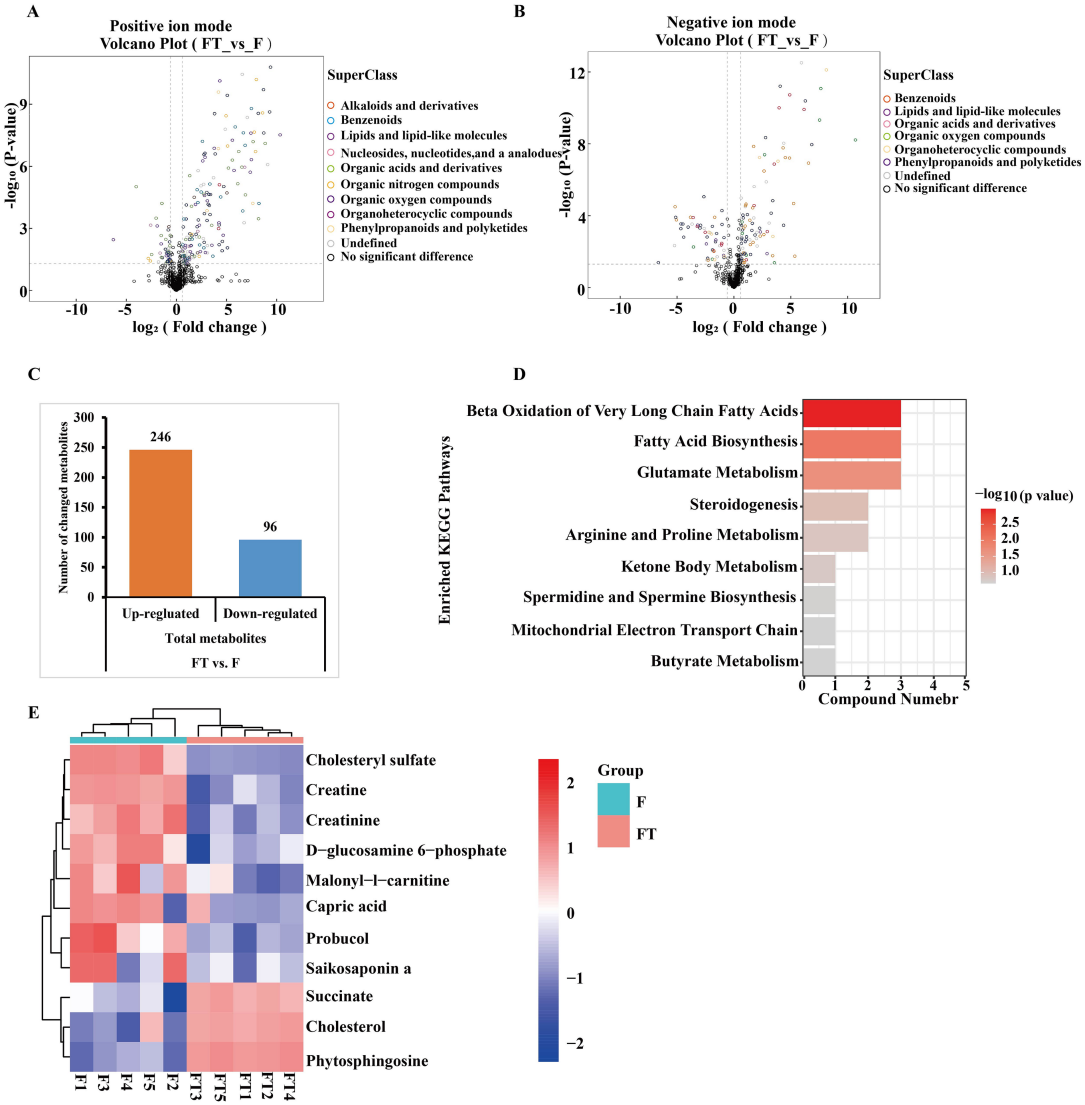
Differential metabolite identification and KEGG pathway enrichment analysis before and after goat sperm freezing. **(A,B)** Positive and negative ion pattern differential metabolite volcano plots. Different colors represent different chemical classifications. **(C)** Total number of differential metabolites identified. Each bar line represents the total number of significant differential metabolites, with orange representing upregulated metabolites and blue representing downregulated metabolites. **(D)** KEGG enrichment pathway map. The vertical axis in the bar graph represents each KEGG metabolic pathway, and the horizontal axis indicates the number of differentially expressed metabolites contained in each KEGG metabolic pathway. The color indicates the *p*-value of enrichment analysis, the darker the color, the smaller the *p*-value and the more significant the enrichment. **(E)** Heatmap of KEGG pathway differential metabolite clustering. FT represents frozen–thawed sperm, and F represents fresh sperm. Each row of the graph represents a differential metabolite (i.e., the vertical coordinate is the significantly differentially expressed metabolite), and each column represents a set of samples (i.e., the horizontal coordinate is the sample information). The color blocks at different positions represent the relative expression of the metabolites at the corresponding positions, red represents relatively high expression, blue represents relatively low expression, and metabolites with close expression patterns are clustered under the same cluster on the left.

**Figure 7:**
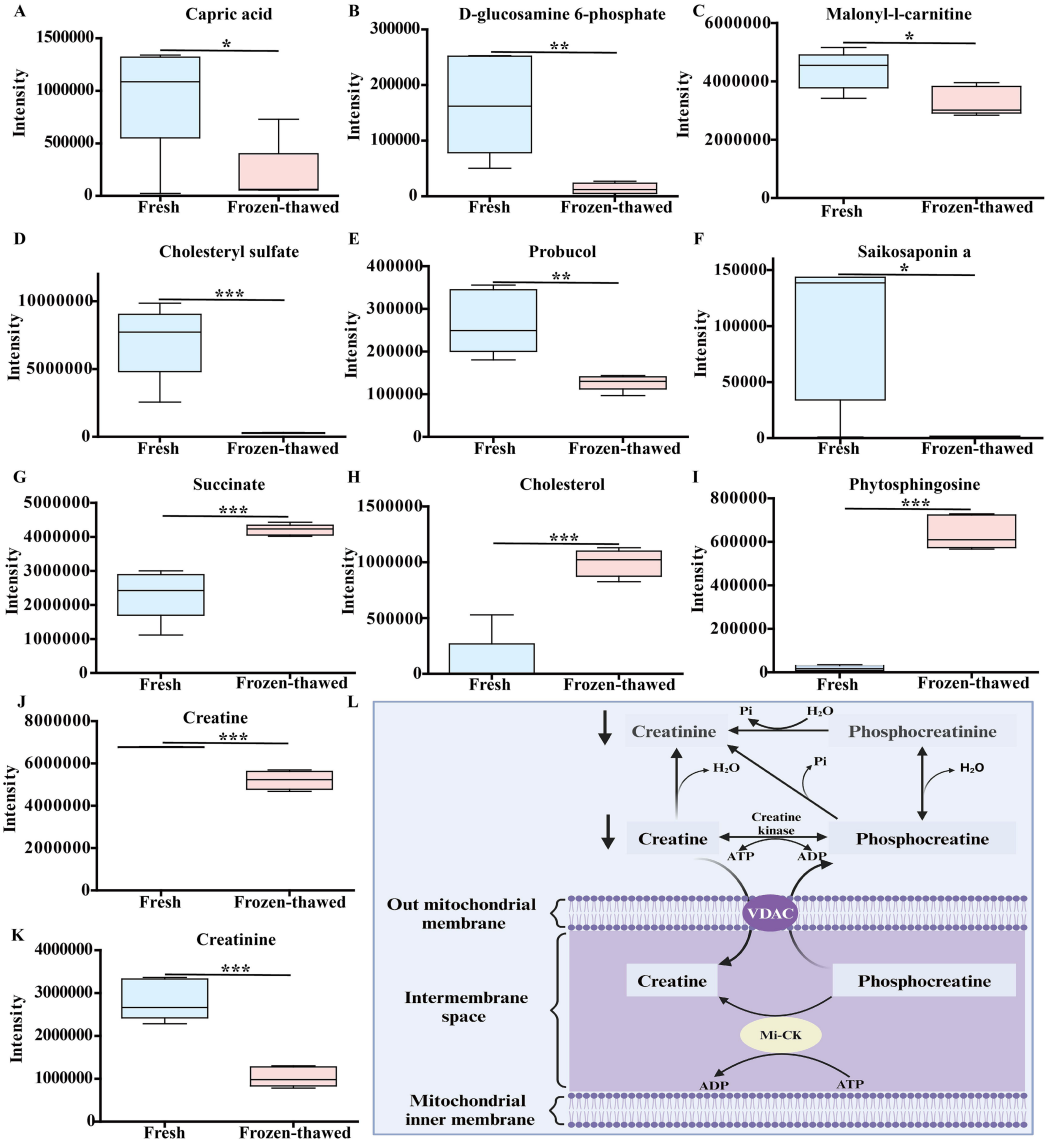
Differences in metabolic profiles between fresh and frozen–thawed sperm. **(A–K)** Metabolomics-based comparative analysis of changes in sperm differential metabolites before and after freezing. The fresh group is labeled in blue and the frozen group in pink, and asterisks indicate *p* < 0.05 (*), *p* < 0.01 (**), and *p* < 0.001 (***). **(L)** Schematic diagram of the relationship between creatinine, creatine, and mitochondrial energy metabolism, where creatinine and water reversibly produce creatine, which dissipates that energy ATP turnover by stimulating the cycling of energy stored in the inner membrane of the mitochondria, mediated by mitochondrial creatine kinase and coupled with the turnover of phosphocreatine (created in Biorender.com). Mitochondrial creatine kinase (Mi-CK); voltage-dependent anion channel (VDAC).

Based on the univariate analysis of the variance method, significant differences were observed in the metabolites of frozen and thawed goat sperm (FC > 1.5 or FC < 0.67, *p* < 0.05). The predominant differential metabolites were identified as lipids and lipid-like molecules (Figures 6A,B). Out of the 342 differential metabolites identified (OPLS-DA VIP > 1, *p* < 0.05), 246 were upregulated and 96 were downregulated (Figure 6C). The Kyoto Encyclopedia of Genes and Genomes (KEGG) pathway analysis revealed that these differential metabolites were mainly enriched in nine metabolic pathways: *β*-oxidation of very long-chain fatty acids, fatty acid biosynthesis, glutamate metabolism, steroidogenesis, arginine and proline metabolism, ketone body metabolism, spermidine and spermine biosynthesis, mitochondrial electron transport chain, and butyrate metabolism (Figure 6D). These nine KEGG metabolic pathways can be classified into two categories based on their relevance to freeze-thawed sperm quality (Figure 6E): energy-related metabolites such as capric acid (Figure 7A), glucosamine-6-phosphate (Figure 7B), malonyl-L-carnitine (Figure 7C), creatine (Figure 7J), creatinine (Figure 7K), and antioxidant-related metabolites such as cholesterol sulfate (Figure 6D), probucol (Figure 7E), and saikosaponin A (Figure 7F) showed a significant decrease, while succinate (Figure 7G), cholesterol (Figure 7H), and phytosphingosine (Figure 7I) exhibited a significant upregulation pattern within other categories. Furthermore, our literature review indicated that creatine is highly expressed in sperm cells, with its synthesis and degradation primarily occurring through spontaneous, non-enzymatic processes (29). Within the cell, ATP, adenosine diphosphate (ADP), creatine, and phosphocreatine must diffuse or be transported through the cell membrane for high-energy phosphate transfer between mitochondria and ATP utilization sites (30). Therefore, the downregulated expression of creatinine and creatine in frozen spermatozoa has negative effects on creatine metabolism (Figure 7L).

### 3.5 Abnormal fluctuations of key rate-limiting enzyme activities in fatty acid biosynthesis and *β*-oxidation metabolism pathways after sperm cryopreservation

**Figure 8:**
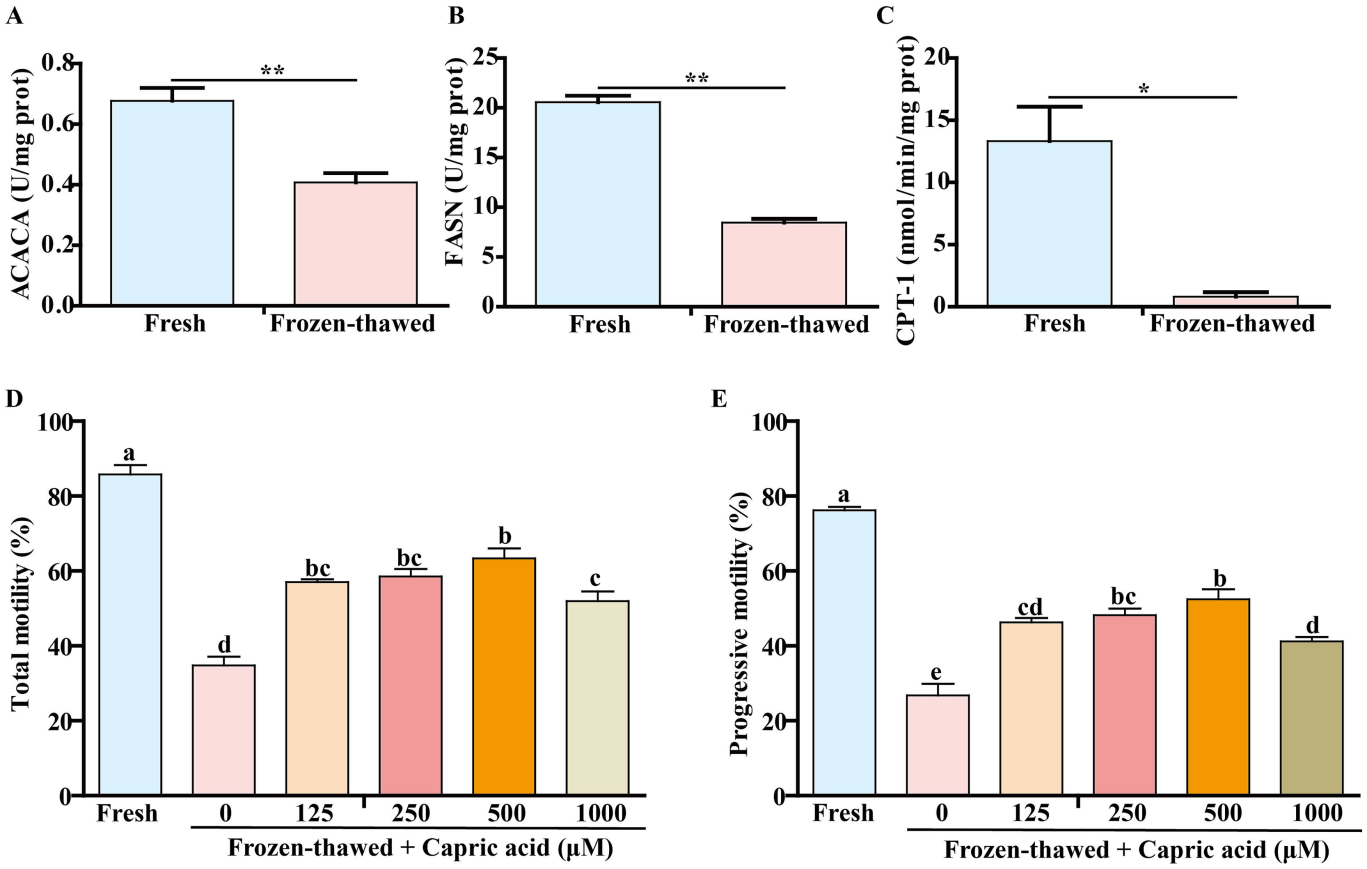
Changes in the activities of key rate-limiting enzymes of metabolic pathways in frozen–thawed sperm and the effect of capric acid on the motility of frozen–thawed sperm. **(A–C)** Changes in ACACA, FASN, and CPT-1 activity before and after sperm freezing. *p* < 0.05 (*) and *p* < 0.01 (**). Effect of adding different concentrations of capric acid to the homemade freezing diluent on the motility of frozen–thawed sperm. **(D)** Total motility. **(E)** Progressive motility. ^a-e^ Within a panel, means without a common superscript differed (*p* < 0.05).

Among the nine metabolic pathways observed in metabolomic analysis, fatty acid biosynthesis and β-oxidation metabolism play crucial roles in sperm energy production (31, 32). Consequently, we conducted further analysis of the activities of the key rate-limiting enzymes ACACA, FASN, and CPT-1 in these metabolic pathways. The activities of ACACA (0.68 ± 0.07 vs. 0.41 ± 0.05, *p* < 0.01) (Figure 8A), FASN (20.57 ± 1.12 vs. 8.46 ± 0.66, *p* < 0.01) (Figure 8B), and CPT-1 (13.32 ± 2.77 vs. 0.81 ± 0.37, *p* < 0.05) (Figure 8C) were significantly lower compared to those of fresh controls.

### 3.6 Capric acid can improve the motility of goat frozen–thawed sperm

Considering that the differentially expressed metabolite capric acid significantly decreased in frozen–thawed sperm and was enriched in the fatty acid synthesis metabolic pathway (Figure 7A), we investigated the effects of various concentrations of capric acid on the motility of frozen–thawed sperm. The results showed that compared to the fresh group, the total motility (85.80 ± 2.51 vs. 34.80 ± 2.29, *p* < 0.001) (Figure 8D) and progressive motility (76.23 ± 0.91 vs. 26.77 ± 3.12, *p* < 0.001) (Figure 8E) of sperm significantly decreased after freezing–thawing, while the addition of 500 μM capric acid significantly improved the total motility (63.40 ± 2.67 vs. 34.80 ± 2.29, *p* < 0.001) (Figure 8D) and progressive motility (52.47 ± 2.62 vs. 26.77 ± 3.12, *p* < 0.001) (Figure 8E) of goat sperm after cryopreservation.

## 4 Discussion

In this study, we initially found that the freezing process resulted in an extremely significant decrease in sperm motility (Figures 2A,B). Furthermore, it was observed that the mitochondrial ultrastructure was impaired following sperm freezing in CBG (Figure 3A). In addition, there was a reduction in ATP content and MMP, accompanied by a significant increase in ROS levels (Figures 3B–D). These findings are consistent with previous studies on mammalian species such as humans (24), pigs (33), horses (34), bulls (35, 36), and sheep (8). This suggests that the detrimental effects of ultra-low temperature freezing on sperm viability are universal across different animal species. Furthermore, it is widely accepted that excessive production of ROS during the freezing process induces oxidative stress (21, 37), which subsequently leads to decreased sperm viability (38). Recent studies have demonstrated that there were two principal sources of intracellular ROS in sperm: one originating from the mitochondria and the other from the plasma membrane (39, 40). ADPH oxidase presented in sperm plasma membrane could be responsible for ROS generation during sperm capacitation (41). The results of our study demonstrated that following the freezing and thawing of the sperm, plasma membrane damage and capacitation-like (cryo-capacitation) changes were observed (Figures 4A–C), as evidenced by complete acrosome detachment or leakage of acrosomal enzymes, similar to natural capacitation, which affect the fertility potential of frozen–thawed sperm.

Given the critical role of sperm mitochondria in energy production and metabolic regulation, we hypothesized that mitochondrial dysfunction may result in disturbances in mitochondrial energy metabolism, which in turn affects the motility of frozen–thawed sperm. To test this hypothesis, we employed metabolomics to explore metabolic profiles of goat freeze-thawed sperm. Previous studies have demonstrated that the differential metabolites between fresh and frozen–thawed semen groups in Dorper sheep include linoleic acid, docosahexaenoic acid, and arachidonic acid, which primarily affect the metabolic pathway of unsaturated fatty acid biosynthesis (25). In Yunshang black goat sperm before and after freezing, the differential metabolites include mannitol, capric acid, and gamma-linolenic acid, which significantly impact the linoleic acid metabolic pathway (27). In Guanzhong dairy goat sperm before and after freezing, sphingolipids, betaine, and choline have been identified as differential metabolites that affect the metabolic pathways of the TCA cycle, unsaturated fatty acids biosynthesis, and sphingolipid metabolism (28). Bull sperm before and after freezing exhibit glycine betaine and pyro-l-glutaminyl-l-glutamine as differential metabolites (26). Our study reveals that the differential metabolites of CBG sperm before and after freezing can be categorized into two main groups: one associated with energy metabolism (capric acid, creatine, and D-glucosamine-6-phosphate), and the other related to the antioxidant activity (saikosaponin A, probucol, and cholesterol sulfate) (Figure 6E). These metabolites predominantly influence fatty acid oxidation and biosynthesis (Figure 6D). It is evident that the changes in differential metabolites along with affected pathways due to ultra-low-temperature freezing negatively impact normal energy metabolism of sperm across different species.

In this study, freezing significantly decreased the activity of ACACA, FASN, and CPT-1 in goat sperm (Figures 8A–C). ACACA catalyzes the conversion of acetyl-CoA to malonyl-CoA (42); FASN is a pivotal enzyme in the endogenous lipogenesis pathway primarily responsible for synthesizing long-chain saturated FA palmitate from acetyl-CoA and malonyl-CoA (43–45), while CPT1 acts as a crucial enzyme initiating the transport of free fatty acids to the mitochondria (31). These three key enzymes play essential roles in fatty acid metabolism (31, 46–48), which generates ATP within the mitochondria to facilitate linear sperm movement (32, 49). Consequently, reduced activity of these three enzymes due to ultra-low temperature freezing undoubtedly impairs sperm motility.

In this study, the expression of antioxidant-related differential metabolites such as saikosaponin A, probucol, and cholesterol sulfate in frozen–thawed sperm was significantly downregulated (Figures 7D–F). Saikosaponin A not only inhibits the overaccumulation of ROS by increasing the activity of antioxidant enzymes but also suppresses inflammation and iron-induced cell death through the PI3K/Akt/Nrf2 pathway (50) or activates the SIRT1/Nrf2 pathway to mitigate oxidative stress in cells (51). Furthermore, it has been demonstrated that saikosaponin A has the capacity to alleviate lipid metabolism disorders in rat liver by stimulating the expression of intracellular lipid and cholesterol catabolism-related genes, including peroxisome proliferator-activated receptor *α* (PPARα), and cholesterol 7α-hydroxylase-1 (CYP7a1) (52, 53). On the other hand, probucol protects cells from peroxide-induced damage directly by activating glutathione peroxidase-1 (54), while cholesterol sulfate enhances resistance against oxidative stress by increasing ATP, lipid, and glycogen content (55). Cholesterol sulfate is a component of cell membranes and has been demonstrated to exert a stabilizing effect, as well as to regulate sperm capacitation (56). Furthermore, it has been demonstrated that it regulates brain energy metabolism through the activation of the Akt/Bcl2 pathway, which reduces the production of ROS, thereby providing neuroprotection (55). Therefore, the decrease in these differential antioxidant-related metabolites after semen freezing in CBG leads to an imbalance in the sperm’s antioxidant system through their respective pathways mentioned above. Consequently, this exacerbates oxidative stress and affects sperm viability (23, 57).

In this study, D-glucosamine-6-phosphate, creatine, and capric acid metabolites associated with energy metabolism exhibited significant downregulation following sperm freezing in goats (Figures 7A,B,J). The diminished metabolites may exert an influence on the synthesis of ATP, which in turn affects the viability of sperm. It has been demonstrated that D-glucosamine-6-phosphate serves as an energy source in the form of phosphosugars (58), while creatine can efficiently generate ATP by connecting ATP-producing sites to subcellular sites of ATP utilization through the creatine/phosphocreatine shuttle system (30). It was reported that SH-SY5Y cells exposed to capric acid for a period of 6 days were demonstrated to have increased activities in several mitochondrial markers, including the activities of citrate synthase and mitochondrial respiratory chain complex I (59). It is also noteworthy that capric acid exposure not only elevates acyl-CoA oxidase 1 activity but also enhances peroxisomal *β*-oxidation of docosanoic acid (60). Furthermore, it has been demonstrated that capric acid can mitigate the oxidative stress induced by cyclophosphamide in IPEC-J2 cells (61). Numerous studies have indicated that capric acid is a potent antioxidant that elicits antioxidant responses through the activation of the Nrf2/ERK1/2 pathway (62–64). Concurrently, capric acid serves as an energy source for brain cells by stimulating glycolysis, which results in the production of lactic acid (65). In this study, we found that the differential metabolite capric acid enriched in goat sperm is involved in the fatty acid synthesis pathway. When 500 μM capric acid was added to the homemade freezing diluent, the motility of cryopreserved goat sperm increased (Figures 8D,E), suggesting that supplemented capric acid may promote ATP production through the above pathway (60), thereby alleviating the decrease in sperm viability induced by freezing.

## 5 Conclusion

**Figure 9:**
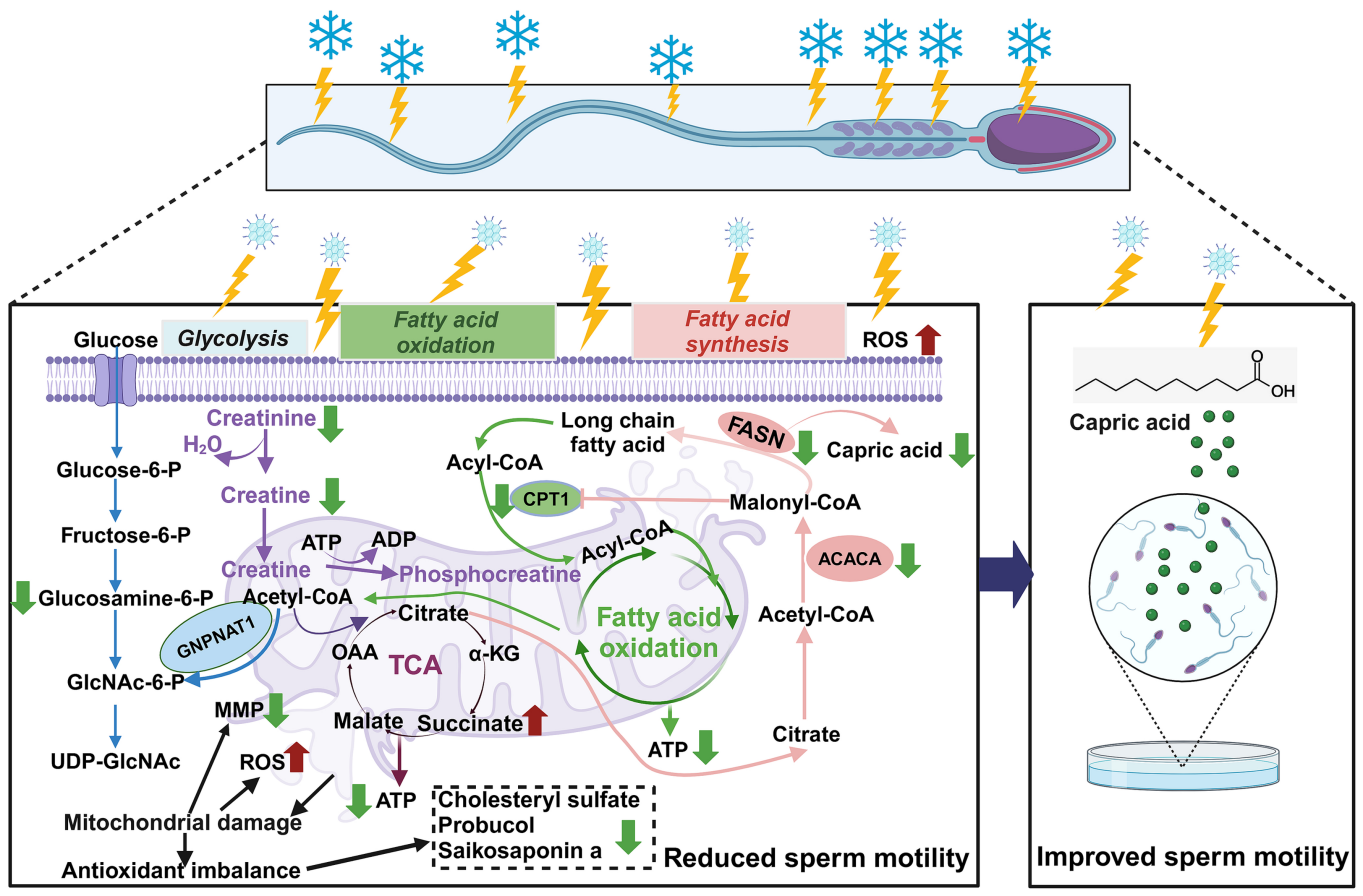
Metabolomics-based analysis of the effects of ultra-low temperature freezing on mitochondrial energy metabolism in goat sperm. Red arrows indicate increases; green arrows indicate decreases. Reactive oxygen species (ROS); mitochondrial membrane potential (MMP); tricarboxylic acid cycle (TCA); adenosine triphosphate (ATP); *α*-ketoglutarate (α-KG); oxaloacetate (OAA); carnitine palmitoyltransferase I (CPT-1); acetyl coenzyme A carboxylase (ACACA); fatty acid synthase (FASN); CPT-1 can be directly inhibited by ACACA direct inhibition by ACACA-produced malonyl coenzyme A. Malonyl coenzyme A is a key intermediate in fatty acid synthesis (FAS) that prevents simultaneous activation of FAS and fatty acid oxidation (FAO) (created in BioRender.com).

Following ultra-low temperature freezing, goat sperm exhibited compromised ultrastructure, disturbed energy metabolism (downregulation of capric acid, D-glucosamine-6-phosphate, and creatine), and antioxidant imbalance (downregulation of saikosaponin A, probucol, and cholesterol sulfate). Moreover, key enzymes involved in the synthesis of long-chain fatty acids and β-oxidation metabolism pathways in the sperm (FASN, ACACA, and CPT-1) demonstrated reduced activity, resulting in abnormal mitochondrial function and decreased sperm motility. Exogenous supplementation of the metabolic intermediate capric acid (500 μM) significantly enhanced the motility of frozen–thawed goat sperm (Figure 9).

## Data Availability

The raw data supporting the conclusions of this article will be made available by the authors, without undue reservation.
